# Early exposure to infections increases the risk of allergic rhinitis—a systematic review and meta-analysis

**DOI:** 10.1186/s12887-023-03870-0

**Published:** 2023-03-01

**Authors:** JunRong Chen, Xiaohua Liu, Zixin Liu, Yaqian Zhou, Li Xie, Jialin Zhang, Jin Tan, Yide Yang, Mei Tian, Yunpeng Dong, Jian Li

**Affiliations:** 1 Key Laboratory of Study and Discovery of Small Targeted Molecules of Hunan Province, School of Medicine, Hunan Normal University, Hunan, 410013 China; 2 Changsha Woman and Children Health Care Hospital Affilated to Hunan Normal University, NO. 416 Chengnan East Road, Changsha, 410007 Hunan China; 3 Third Xiangya Hospital, Central South University, 138 Tongzipo Road, Changsha, 410013 Hunan China; 4 Second Xiangya Hospital, Central South University, 139 Renmin Road, Changsha, 410011 Hunan China; 5 Key Laboratory of Molecular Epidemiology of Hunan Province, School of Medicine, Hunan Normal University, Hunan, 410081 China; 6 Department of Otolatyngoloty-Head and Neck Surgery, the First College of Clinical Medical Science, Yichang Central People’s Hospital, Three Gorges University, Hubei, 443000 China; 7 Key Laboratory of Model Animals and Stem Cell Biology in Hunan Province, School of Medicine, Hunan Normal University, Hunan, 410013 China

**Keywords:** Infections, Children, Allergic rhinitis, Meta-analysis

## Abstract

**Objective:**

The purpose of this study was to provide evidence for early life care by meta-analyzing the relationship between infection during pregnancy and up to 2 years of age and the risk of subsequent allergic rhinitis (AR).

**Methods:**

Published studies up to April 2022 were systematically searched in PubMed, Embase, Web of Science, Cochrane Library, SinoMed, CNKI, Wanfang Database, and VIP. Literature screening, including quality assessment, was performed, and the effect values (*OR, HR, RR*) and 95% confidence intervals (95% CI) of infection during pregnancy and up to 2 years of age and allergic rhinitis were extracted from each qualified study.

**Results:**

In total, 5 studies with a sample size of 82,256 reported the relationship between infection during pregnancy and offspring AR. Meta-analysis showed that maternal infection during pregnancy was associated with an increased risk of childhood AR in offspring (*OR* = 1.34, 95% CI: 1.08–1.67). Altogether, 13 studies with a sample size of 78,426 reported evidence of an association between infection within 2 years of age and subsequent AR in children. A pooled meta-analysis of all studies showed that early infection within 2 years of age was closely associated with childhood AR (OR = 1.25, 95% CI: 1.12–1.40), especially upper respiratory tract infection (*OR* = 1.32, *95% CI*: 1.06–1.65) and gastrointestinal infections (*OR* = 1.37, 95% CI: 1.01–1.86), but ear infection showed similar results in the cohort study (*OR* = 1.13, *95% CI*: 1.04–1.22).

**Conclusion:**

Current evidence suggests that infection during pregnancy, early upper respiratory infection, gastrointestinal infections and ear infection within 2 years of age would increase the risk of AR in children. Therefore, the prevention of infection during pregnancy and in infancy and young children needs to be emphasized.

**Supplementary Information:**

The online version contains supplementary material available at 10.1186/s12887-023-03870-0.

## Introduction

One of the most common chronic diseases, especially in children, is allergic rhinitis (AR). There is a chronic immune-mediated disease mediated by immunoglobulin E that occurs on the nasal mucosa after a specific individual is exposed to allergens, and symptoms include sneezing, watery mucus, nasal itching and congestion [1], which seriously affect children's quality of life, daily activities, sleep and learning [2]. According to a meta-analysis covering 102 countries, the worldwide prevalence of childhood AR is 12.66% [3].

However, a comprehensive analysis of the relationship between infection and AR is lacking despite the fact that its etiology is unclear, it is currently believed to be closely related to the combination of genetic and environmental factors. In addition to genetic and epigenetic mechanisms, the living environment and gut microbiota also affect AR. In accordance with the Developmental Origins of Health and Diseases (DOHaD) theory, adverse exposures during early life may have an adverse impact on the development of programming and the occurrence of chronic diseases in late life. However, taking into account the hygiene hypothesis, early exposure to microorganisms may prevent the development of allergic diseases. The better your lifestyle, the less likely you are to encounter microorganisms, leading to a greater incidence of allergic diseases. The period of pregnancy is the most critical period for the development of the fetus, and mounting evidence has shown that maternal infection during pregnancy increases the risk of adverse perinatal outcomes and long-term health outcomes for offspring, such as low birth weight and mental illness [4–6]. The perturbed gut microbiota in the first 1000 days of life, from pregnancy to 2 years after birth, increases the risk for allergic disease and obesity in later life, highlighting the importance of understanding the relationships of perinatal factors with the establishment of diverse gut microbiota [7–10].

Although previous studies have shown that maternal infection during pregnancy can increase the risk of asthma and eczema in offspring [11], a meta-analysis by Van Meel et al. [12] also showed that early respiratory tract infection was associated with the development of asthma in school-age children, but the relationship between infection and AR has received less attention.

Recently, an increasing number of studies have surveyed the relationship between early exposure to infections during pregnancy and within 2 years old and the risk of AR in late life. Recent studies have shown that the occurrence of AR is closely related to exposure to antibiotics and air pollution in early life [13, 14]. Of interest, there was a lack of uniformity in the research conclusions on the relationship between infections and later AR. McKeever [15] showed that early personal infections do not provide significant protection against allergic diseases, whereas Bremner found that early respiratory infections may increase the risk of later allergic rhinitis [16]. As early exposure to infection might be an understanding of the pathogenesis of AR, it is necessary to synthesize all available published literature on the relationship between infection during the early 1000 days of life and the risk of AR in late life in the form of a comprehensive meta-analysis.

## Methods

### Data sources and search strategy

The search strategies used the PICO principle to ensure that the retrieved journal-published literature was as comprehensive as possible. Search terms were including medical subject heading terms and text words related to subjects were developed in Pubmed and then adapted for Pubmed, Embase, Web of science, Cochrane, Sinomed, CNKI, Wanfang Database, and VIP from inception through April 30, 2022,the search terms were as follows:antenatal, prenatal, pregnancy, pregnant, perinatal, gestational, maternal, mother, newborn, infant, early life, toddler, febrile, infection, infestation, rhinitis, allergic, rhinitis, allergic, seasonal, allergic rhinitides, hayfever, hay fever. No publication, population, or language restrictions were applied, and attention was paid to checking the list of references on relevant topics. In addition, it was impossible to contact authors to have access to the full text or original data. Search terms and strategies are described in Additional file 1.

### Study selection

Titles and abstracts of all potentially eligible articles retrieved from each database and managed in Endnote X9 software. The literature criteria employed for the meta-analysis included the following: (1). Study population: children aged 0–18 years old. (2). Study types: cohort studies, cross-sectional studies, and case–control studies. Exposure factors: infection within 2 years after birth or maternal infection during pregnancy. (4). Outcome: clearly the offspring have AR, the relative risk (RR), hazard ratio (HR), or odds ratio (OR) and their confidence intervals can be obtained, or enough data to calculate them. The exclusion criteria were as follows: (1). The full text or original data are not available. (2). Publication languages other than Chinese or English. (3). Duplicate publications. (4). Reviews, systematic reviews or meta-analyses, conference abstracts, research protocols. (5). Viral skin infections (only one study). (6). Low-quality research.

### Quality assessment and data extraction

The quality of the cohort studies and case–control studies was measured with the Newcastle–Ottawa Scale (NOS) [17], and cross-sectional studies were assessed using the Agency for Healthcare Research and Quality [18] (AHRQ). The NOS scale has a total score of 9 points, including three aspects: study population selection, comparability, and outcome, of which comparability can be scored up to 2 points, and studies with scores < 5 points are considered high risk of bias studies. The AHRQ scale has a total score of 11 points with 11 items, answering “yes” can be scored as 1 point, and answering “unclear or no” is scored as 0 points. The quality scale is divided into 0–3 points for low quality, 4–7 points for medium quality, and ≥ 8 points for high quality. In this study, the quality of literature assessed as moderate to high quality was included in the analysis (Score ≥ 6 points).

The parameters and data were extracted using a standardized spreadsheet from each study, including author, year, study country, study type, study object, sample size, exposure factors, diagnostic method, and effect values. Two researchers independently screened the literature and extracted data according to the selection of the study. After each phase of the screening and data extraction process, any disagreements regarding records between two researchers were resolved by discussion or by consulting a third investigator if consensus could not be reached.

### Data synthesis and analysis

RevMan 5.4 and Stata 16.0 software were used for statistical analysis, and the *OR* and 95% confidence interval (95% CI) used in the meta-analysis *P* ≤ 0.05 was considered to be statistically significant. Statistical heterogeneity across studies was tested by the *Q* statistic and* I*^*2*^ value. If no significant heterogeneity was examined (*I*^*2*^ < 50% and *P* > 0.1), pooled estimates were calculated using a fixed‐effects model; otherwise, a random‐effects model was adopted [19, 20]. Analysis of the risk relationship between different infections and AR in children. First, if a study reported different exposures;Second, if the study was stratified by exposure factors and study participants (number of infections, different periods of infection, and different ages of children) without providing overall estimates of infection and AR,the effect estimate and 95% *CI* from the literature were combined at first and as the final extracted effect into meta-analysis. Moreover, a subgroup analysis was performed,and sensitivity analysis was estimated by omitting every study individually. If the heterogeneity among the studies decreases after excluding one study, it shows that this study is the cause of the heterogeneity. Publication bias was assessed using funnel plots and Begg’s test of bias, with *P* < 0.05 indicating significant publication bias.

## Results

### Study selection and characteristics

**Fig. 1:**
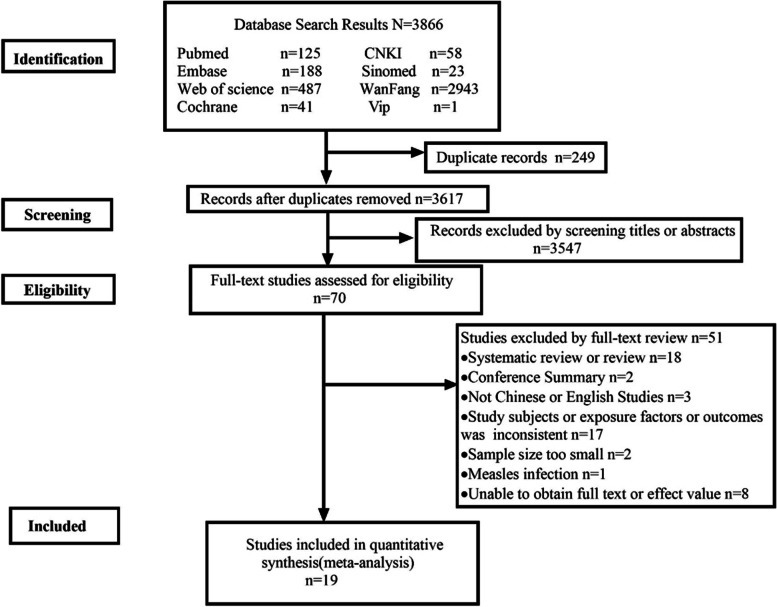
Screening flow chart

In total, 3866 studies were identified according to our search strategies. A total of 249 duplicate studies were excluded, and 3547 studies were inconsistent with the research purpose after reviews of the titles and abstracts. Then, 70 studies were screened and required full-text reading for detailed evaluation. Finally, 19 studies were included in this meta-analysis after full text review. More specific screening details are shown in Fig. 1 and totally 51 references were excluded and excluded reasons are shown in the Additional file 2. Among these 19 studies, 14 studies with a total sample size of 78,426 reported evidence of infection within 2 years of age and subsequent AR in children, including 7 cohort studies [15, 21–26], 5 cross-sectional studies [27–31] and 2 case–control studies [16, 32]. Five studies with a total sample size of 82,256 reported evidence of infection during pregnancy and AR in offspring, including 3 cohort studies [33–35], 1 cross-sectional study [36] and 1 case–control study [37]. Except for the study of Thomson 2010 [21], the quality evaluation of all the included studies was of medium to high quality. This study used follow-up parental reports for infection exposure and AR outcome determinations. The following analysis did not include this evidence because the assessments were unreliable and the sample size was not large enough. Three of the remaining 18 studies extracted crude effect sizes [22, 27, 33], one was obtained by calculation [27], and all others extracted adjusted effect sizes. Tables 1, 2 and 3 summarize the specific information of the included studies and quality evaluation, respectively.

**Table 1:** Characteristics of included studies

Author Year	Country	Exposure period	N	Age (y)	Exposure factors	Outcome evaluation
**Mckeever 2002 **[34]	United Kingdom	During pregnancy	29238	0–11	①	a
**Hsieh 2016 **[35]	Taiwan, China	During Pregnancy	42217	0–9	②	a
**Illi 2014 **[33]	Germany	During Pregnancy	526	0–5	③	c
**Xu 1999 **[36]	Finland	During Pregnancy	8088	7	④	c
**Liao 2015 **[37]	China	During Pregnancy	2187	4–12	⑤	a
**Bremner 2008 **[16]	United Kingdom	First year of life	3549	2–5	⑥⑦⑩	Not described
**De 2005 **[28]	Netherlands	From birth to 2 years of age	1555	8–13	⑥	b
**Mckeever 2002 **[15]	United Kingdom	First year of life	29238	0–8	⑥⑦⑧	b
**Kang 2019 **[32]	China	From birth to 6 months old	500	12.8 ± 5.2, 13.7 ± 5.2	⑥⑦	b
**Lin 2015 **[23]	Taiwan, China	First month of life	16413	0–8	⑩	a
**Ramsey 2007 **[22]	United States	First year of life	440	0–7	⑥⑨	a
**Kemp 2009 **[29]	Australia	First month of life	913	8,16	⑥	a
**Macintyre 2010 **[24]	Germany	From birth to 2 years of age	1690	6	③	b + SPT/IgE
**Mai 2009 **[25]	Sweden	First year of life	3306	0–4、0–8	⑥⑧	c
**Thomson 2010 **[21]	Australia	From birth to 2 years of age	488	0–6	⑥⑦⑧	b
**Kitsantas 2018 **[30]	United States	First year of life	1466	6	⑥	c
**Ponsonby 1999 **[31]	Australia	From birth to 85 days old	865	7	⑥	b
**Strachan 1996 **[27]	United Kingdom	First month of life	17968	3–16	⑥⑦	c
**Harris 2007 **[26]	United Kingdom	From birth to 2 years of age	523	0–8	⑥⑦⑨⑩	b

①: gastrointestinal, respiratory, conjunctivitis, otitis media, candida, bacterial,viral, ②: periodontitis and gingivitis, ③: colds, ④: febrile infections, ⑤: bacterial and viral, ⑥: Respiratory infection, ⑦: Gastrointestinal infection, ⑧: Otitis media infection, ⑨: Ear infection, ⑩: Urinary infection

a: Doctor's diagnosis, b: ISAAC:International study of asthma and allergies in childhood, c: Self-report, SPT:Skin prick test

**Table 2:** Quality assessment of included studies—cohort study and case–control study

Author Year	Study population selection	Intergroup comparability	Outcome measurement	Total points
Mai 2009 [25]	4	1	2	7
Lin 2015 [23]	4	2	2	8
Thomson 2010 [21]	2	1	2	5
Mckeever 2002 [15]	4	2	2	8
Macintyre 2010 [24]	4	2	1	7
Illi 2014 [33]	4	1	2	7
Ramsey 2007 [22]	3	1	2	6
Hsieh 2016 [35]	4	2	3	9
Mckeever 2002 [34]	4	2	2	8
Kang 2019 [32]	4	1	1	6
Bremner 2008 [16]	3	2	2	7
Liao 2015 [37]	4	1	1	6
Harris 2007 [26]	4	2	2	8

**Table 3:** Quality assessment of included studies—cross-sectional study

Author Year	1	2	3	4	5	6	7	8	9	10	11	Total points
De 2005 [28]	Y	N	Y	N	Y	N	Y	Y	N	Y	N	6
Kitsantas 2018 [30]	Y	N	Y	N	Y	N	N	Y	Y	Y	N	6
Ponsonby 1999 [31]	Y	Y	Y	N	Y	N	N	N	Y	Y	N	6
Kemp 2009 [29]	Y	N	Y	N	Y	Y	Y	Y	N	Y	N	7
Xu 1999 [36]	Y	N	Y	Y	Y	N	N	Y	N	Y	N	6
Strachan 1996 [27]	Y	N	Y	N	Y	N	Y	Y	N	Y	N	6

Y:Yes, N:No/Unclear; 1. Define the source of information (survey, record review); 2. List inclusion and exclusion criteria for exposed and unexposed subjects (cases and controls) or refer to previous publications; 3. Indicate time period used for identifying patients; 4. Indicate whether or not subjects were consecutive if not population-based; 5. Indicate if evaluators of subjective components of study were masked to other aspects of the status of the participants; 6. Describe any assessments undertaken for quality assurance purposes (eg, test/retest of primary outcome measurements; 7. Explain any patient exclusions from analysis; 8. Describe how confounding was assessed and/or controlled; 9. lf applicable, explain how missing data were handled in the analysis; 10. Summarize patient response rates and completeness of data collection; 11. Clarify what follow-up, if any, was expected and the percentage of patients for which incomplete data or follow-up was obtained

### Meta-analysis of infection during pregnancy and the risk of AR

**Fig. 2:**
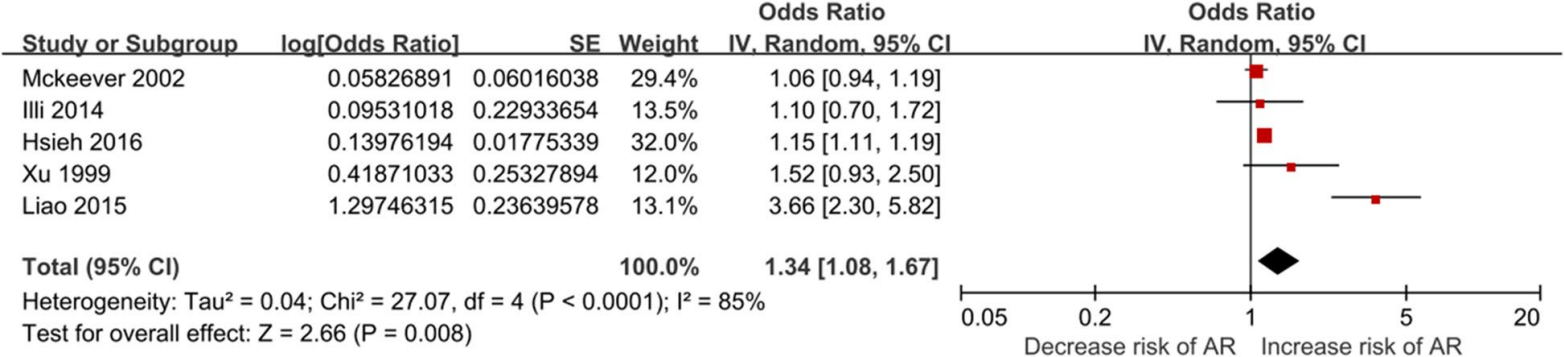
Meta-analysis of infection during pregnancy and the risk of AR

A total of 5 studies identified the relationship between maternal infection during pregnancy and the risk of AR in offspring. Maternal infection during pregnancy was associated with an increased risk of AR in offspring (*OR* = 1.34, 95% CI: 1.08–1.67) in all 5 studies as a forest plots (Fig. 2). In performing subgroup analyses, infection during pregnancy was associated with a significantly increased risk of AR in offspring in 3 cohort studies (*OR* = 1.14, *95% CI*: 1.10–1.18) and in the literature that AR was diagnosed by a doctor (*OR* = 1.38**,** *95% CI*: 1.06–1.81**)**, but there was a trend in 2 case–control studies (*OR* = 2.37, *95% CI*: 1.00–5.61) and in the literature that AR was diagnosed by self-reporting (*OR* = 1.38**,** *95% CI*: 1.06–1.81**).** The analysis results are as follows (Table 4).

**Table 4:** Subgroup analysis of infection during pregnancy and the risk of AR

	*OR*(95%*CI*)	*P*	Heterogeneity	
Total studies	**1.34 (1.08–1.67)**	**0.008**	85%	*P* < 0.001
Study type				
Cohort study	**1.14(1.10–1.18)**	**< 0.001**	0%	0.42
Non-cohort study	**2.37 (1.00–5.65)**	**0.05**	84%	*P* < 0.001
Outcome evaluation				
Doctor's diagnosis	**1.38 (1.06–1.81)**	**0.02**	92%	*P* < 0.001
Self-report	1.27 (0.91–1.78)	0.16	0%	0.34

### Meta-analysis of infection within 2 years of age and the risk of AR

**Fig. 3:**
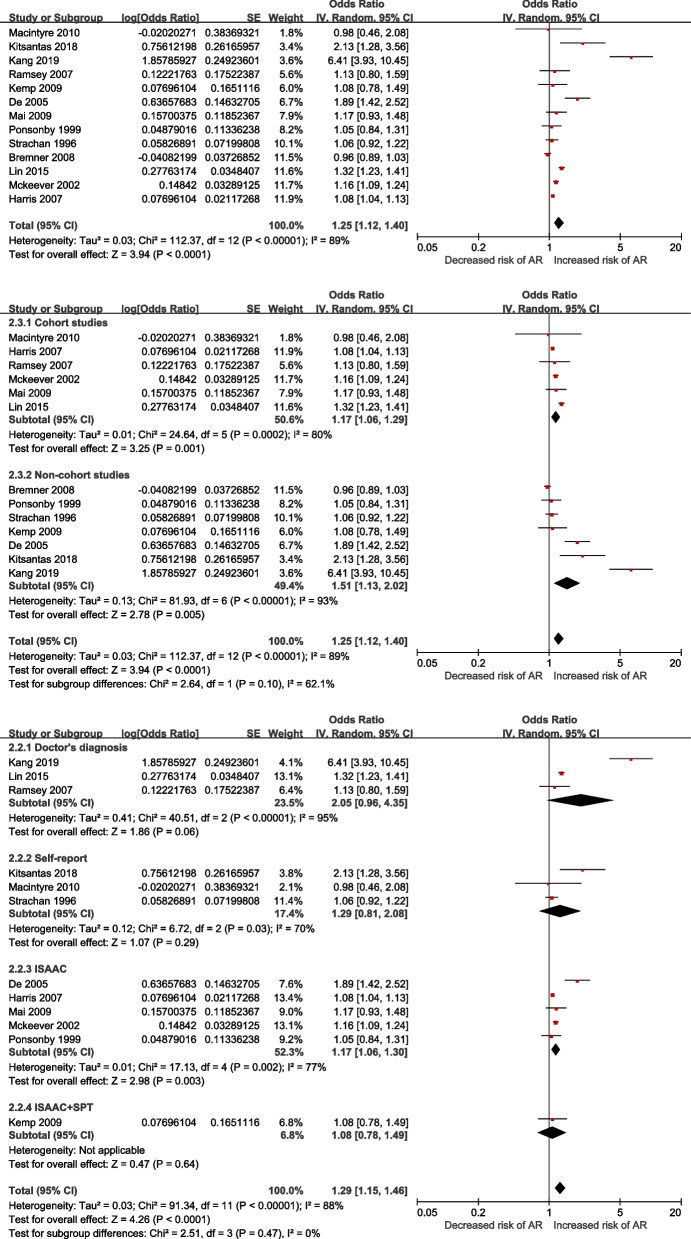
Meta-analysis of infection within 2 years of age and the risk of allergic rhinitis

These overall results showed that early infection within 2 years of age was closely associated with childhood AR (*OR* = 1.25, *95% CI*: 1.12–1.40), especially in cohort studies (*OR* = 1.17, *95% CI*: 1.06–1.29) and noncohort studies (*OR* = 1.51, *95% CI*: 1.13–2.02). Compared with 3 papers that confirmed AR by self-reporting, the results showed that early infection within 2 years of age was significantly related to AR in outcome evaluated by ISAAC (*OR* = 1.17, 95% CI: 1.06–1.30**)** and in trend related to AR in outcome evaluated by doctor's diagnosis (*OR* = 2.05, *95% CI*: 0.96 -4.35) (Fig. 3).

Early upper respiratory tract infection, bronchitis, lower respiratory tract infection, ear infection, gastrointestinal infection and neonatal urinary tract infection were reported in the included studies. Subtype of infection analysis showed that a history of upper respiratory tract infection was associated with an increased risk of AR in children (*OR* = 1.32, 95% CI: 1.06–1.65). However, neither a history of bronchitis nor lower respiratory tract infection was related to AR in children. Ear infection was associated with an increased risk of AR in children in 4 cohort studies (*OR* = 1.13, *95% CI*: 1.04–1.22) but not in the case–control study (*OR* = 0.96, *95% CI*: 0.87–1.06). Gastrointestinal infection increased the risk of AR in children in the cohort study (*OR* = 1.20, 95% CI: 1.05–1.37), but there was an obvious tendency in 3 noncohort studies (*OR* = 1.70, 95% CI: 0.95–3.06, *p* = 0.08). Urinary tract infection was not associated with an increased risk of AR in children in any type of study. The specific results are shown in Table 5.

**Table 5:** Subgroup analysis of infection within 2 years of age and the risk of allergic rhinitis

	No	*OR* (95%*CI*)	*P*	Heterogeneity	
All studies	13	**1.25 (1.12–1.40)**	**< 0.001**	*I*^*2*^ = 89% *P* < 0.001	
Cohort studies	6	**1.17 (1.06–1.29)**	**0.001**	*I*^*2*^ = 80% 0.002	
Non-cohort studies	7	**1.51 (1.13–2.02)**	**0.005**	*I*^*2*^ = 93% *P* < 0.001	
Outcome evaluation					
Doctor's diagnosis	3	2.05 (0.96 -4.35)	0.06	*I*^*2*^ = 95% *P* < 0.001	
Self-report	3	1.29 (0.81–2.08)	0.29	*I*^*2*^ = *70%* 0.03	
ISAAC	5	**1.17 (1.06–1.30)**	**0.003**	*I*^*2*^ = 77% 0.002	
ISAAC + SPT	1	1.08 (0.78–1.49)	0.64	NA NA	
Upper respiratory tract infection					
All studies	7	**1.32 (1.06–1.65)**	**0.01**	*I*^*2*^ = 83%	*P* < 0.001
Cohort study	1	**1.22 (1.05–1.41)**	**0.008**	NA	NA
Non-cohort studies	6	**1.37 (1.02–1.85)**	**0.02**	*I*^*2*^ = 85%	*P* < 0.001
Bronchitis					
All studies	3	0.98 (0.88–1.09)	0.74	*I*^*2*^ = 47%	0.15
Cohort studies	2	1.32 (0.87–2.00)	0.97	*I*^*2*^ = 41%	0.19
Case control study	1	0.96 (0.86–1.07)	0.46	NA	NA
Lower respiratory tract					
All studies	5	1.16 (0.79–1.70)	0.46	*I*^*2*^ = 75%	0.003
Cohort studies	2	1.03 (0.63–1.68)	0.9	*I*^*2*^ = 0%	0.89
Non-cohort studies	3	1.24 (0.71–2.16)	0.45	*I*^*2*^ = 87%	0.003
Ear infection					
All studies	5	1.06 (1.00–1.13)	0.06	*I*^*2*^ = 39%	0.16
Cohort studies	4	**1.13 (1.04–1.22)**	**0.003**	*I*^*2*^ = 0%	0.97
Case control study	1	0.96 (0.87–1.06)	0.42	NA	NA
Gastrointestinal infection					
All studies	4	**1.37 (1.01–1.86)**	**0.04**	*I*^*2*^ = 91%	*P* < 0.001
Cohort study	1	**1.20 (1.05–1.37)**	**0.007**	NA	NA
Non-cohort studies	3	1.70 (0.95–3.06)	0.08	*I*^*2*^ = 94%	*P* < 0.001
Urinary tract infection					
All studies	2	1.16 (0.83–1.63)	0.39	*I*^*2*^ = 70%	0.07
Cohort study	1	1.32 (1.23–1.41)	< 0.001	NA	NA
Case control study	1	0.92 (0.63–1.35)	0.67	NA	NA

### Sensitivity analysis and publication bias test

**Fig. 4:**
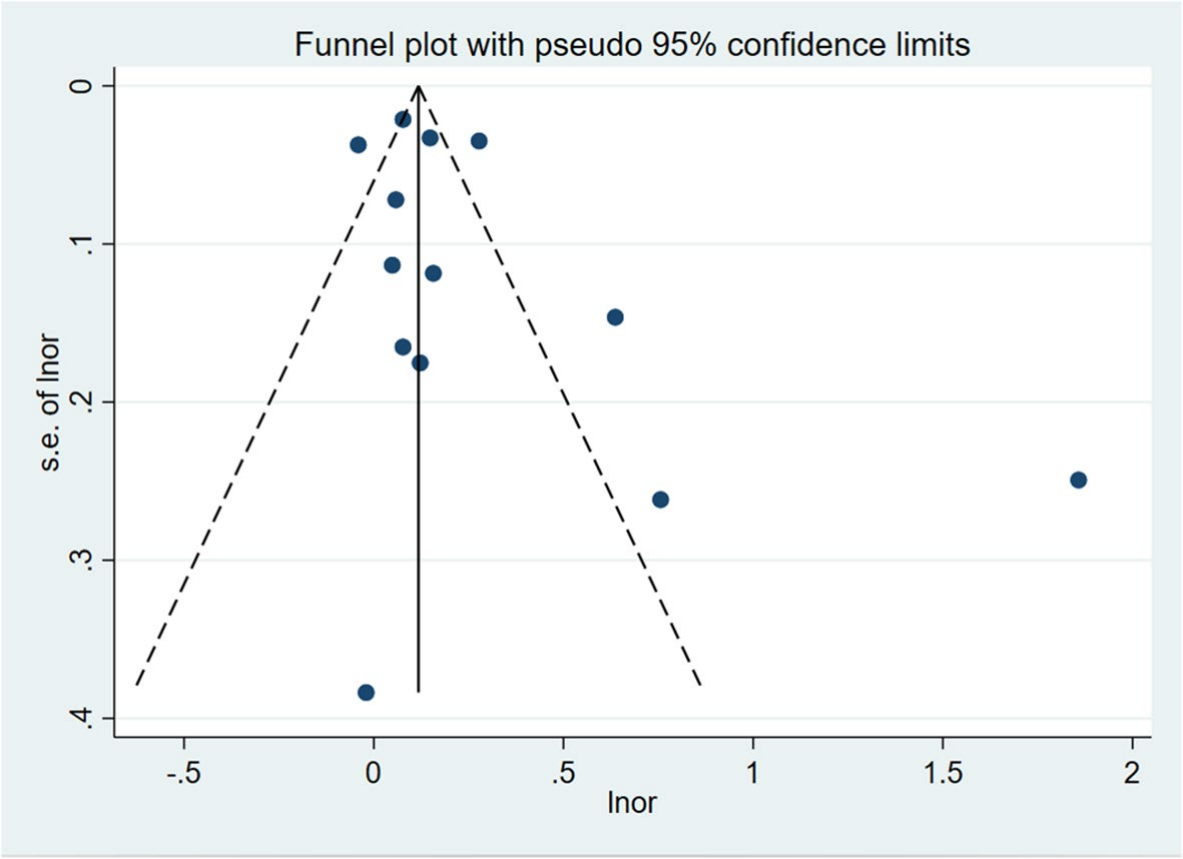
Funnel plots

For maternal infection, the overall sensitivity analysis was carried out by removing the literature one by one, and it was found that the effects on the combined results were stable and reliable. However, when Liao 2015 [37] was removed, the remaining heterogeneity between studies became no longer significant (*I*^*2*^ = 0%, *P* = 0.39), and the results still showed that infection during pregnancy could increase the risk of developing AR in offspring (*OR* = 1.14, 95% CI: 1.11–1.18). Funnel plots did not show the possibility of publication bias, and Begg’s test determined this outcome (*P* = 0.451, *P* > 0.05) (Fig. 4). Publication bias test was not performed in this analysis because fewer than 10 studies were included [38].

**Fig. 5:**
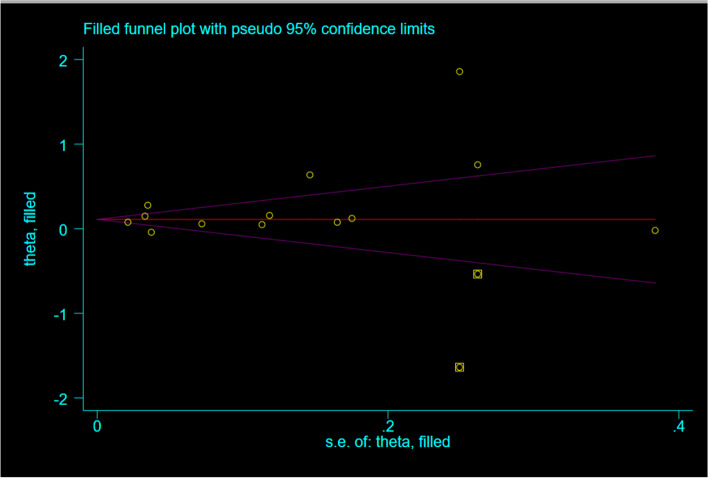
Filled funnel plot

For another infection in the first two years of life, based on the results of the above preliminary analysis, the sensitivity analysis was carried out by using the method of eliminating literature one by one. It can be seen that unstable results for the upper respiratory tract infection and greater heterogeneity between studies, when De 2005 [28] and Kang 2019 [32] studies were excluded, a significant decrease of the test for heterogeneity was observed (*I*^*2*^ = 0*%*,*P* = 0.02), the results of all remaining studies still show that upper respiratory tract infection was associated with increased risk of AR in children (*OR* = 1.11,*95%CI*:1.02–1.22). Similarly, for the gastrointestinal tract, after excluding the study of Strachan 1996 [27], the results of the remaining three studies were (OR = 1.59, 95% CI: 1.06–2.37). The sensitivity analysis results of other factors are stable and reliable. As before, no evidence of a significant publication bias was found, which was confirmed by the Begg’s test (*P* = 0.246, *P* > 0.05). However, asymmetry can be found in funnel plots (Fig. 4). In order to solve this problem, we estimated a sensitivity analysis using the trim and fill method (adding 2 new virtual studies) showed that the results was reliable (OR = 1.15,95%CI:1.01–1.30). Thus, we found that the outcome was not affected by publication bias. (Fig. 5).

## Discussion

In the current study, we shed light on the link between infection and AR in children. The results of the stratified analysis found that maternal infections during pregnancy as well as early infections of the upper respiratory tract, gastrointestinal infections and ear infection within 2 years old increased the risk of AR in children.

AR is more prevalent in children than any other chronic illness, which makes it a serious public health concern. Taking preventive measures requires a thorough understanding of the risk factors for allergic diseases in children. It is increasingly recognized that the development of fetal and infant allergies is influenced in large part by the early years of their life, and this cannot be ignored. During the past few years, the DOHaD theory has become a hotspot in the research field of allergic diseases. Maternal infection during pregnancy can increase the risk of asthma and eczema in offspring [11]. A meta-analysis [12] also showed that early respiratory tract infection within 2 years old was associated with the development of asthma in school-age children. It has been demonstrated in numerous studies that maternal adverse exposure during pregnancy, such as passive smoking [39], diet [40], psychological status [41], pregnancy complications [42], and antibiotic exposure during pregnancy [43], was associated with a greater likelihood of AR in offspring. Additionally, studies have provided compelling evidence that adverse exposures in the early postnatal period, such as antibiotic use [13], pet exposure [44] and air pollution [45], may increase the risk of a child developing allergies. This meta-analysis demonstrated that infection during the first 1000 days of life from pregnancy to 2 years of age could increase the subsequent childhood AR.

To the best of our knowledge, some of these correlation results may be partly explained by the fact that infections can alter microbiome stability. It has been suggested that the balance of gut and lung microbes may play a role in allergic disease [46]. Studies have shown that AR patients have fewer gut microbes than healthy individuals [47]. As a result of these arguments, we may be able to conclude that the microbiota may play a role in allergic diseases. The microbial composition of the nasopharynx has been shown to influence airway sensitivity in a recent study [48]. Similarly, infection during pregnancy can lead to the presence of bacteria in the uterus or amniotic fluid, which can be transmitted to the fetus and then affect the gut flora of the fetus [49]. According to Gayen et al. [50], the control of inflammatory, immune, and respiratory processes was influenced by differential leukocyte gene expression in neonates who were exposed to fetal membrane infection during pregnancy. In animal studies, elevated IL-17A production has been associated with allergic airway inflammation in neonates infected with Streptococcus pneumoniae [51]. The latest study also showed that mice infected with Streptococcus pneumonia developed more pronounced airway responses and had a higher level of serum-specific IgE and Th2 cytokines in the lung. It has therefore been shown that early respiratory infection with Streptococcus pneumoniae can exacerbate later allergic airway inflammation and adult-associated asthma caused by house dust mites [52].

It is worth mentioning that research on the effects of antibiotic use on allergic diseases has gradually increased. It has been noted that both exposure to antibiotics and infections have been shown to be related to allergic diseases in the absence of mutual adjustment factors [31]. Slightly inconsistent with this opinion, Mai et al. [53] noted that early postnatal respiratory infection may confound the association between antibiotic use and allergic diseases. McKeever et al. [34] examined the interaction of infection and antibiotic use during pregnancy with allergic disease in offspring in two models simultaneously. According to their findings, infections are not associated with AR in offspring, although adjusting it did not notably affect the use of antibiotics, increasing the risk of allergic disease. However, Lin et al.'s study [54] found that both the initial infection and antibiotic use are independent risk factors for secondary atopic dermatitis in children. Thus, future research should examine whether infections and AR are related, whether the correlation could be confounded by antibiotic use, and whether antibiotics are related to these conditions.

The strength of this study lies in the fact that a search of eight databases was conducted for this study, and the quality of the included literatures were rated as moderate to high. The shortcoming of this study: there is heterogeneity among evidence, and clinically, heterogeneity exists first, as an example, De et al. [28], was a cross-sectional study with a selected specific population aged 8–13 years, and a recall questionnaire provided the main evaluation method. Then, the large majority of reports were from populations of predominantly European countries, which would lack the accuracy of interpretation of the overall population. Second, methodological differences could also contribute to the divergence of results between studies, as different adjusted confounding factors among the studies, especially most studies did not examine the impact of antibiotic use following infection. In addition, with the exception of cohort studies, outcome measures obtained through self-report or information from parental interviews may be liable to recall bias. Furthermore, very few studies have been conducted on the impact of pregnancy infections and urinary tract infections on AR, and there was no clear explanation for pregnancy infection. In this analysis, the 5 studies addressed different types of infection, and more prospective cohort studies should ideally be designed with a greater focus on the confounding effects of infection and antibiotic use in the future.

## Conclusion

In conclusion, our study identifies and characterizes that infection during pregnancy, early upper respiratory infection, gastrointestinal infections and ear infection within 2 years of age is associated with subsequent childhood AR and suggests that health care workers should strengthen health education in their communities. However, the confounding effects of antibiotics and infection remain to be studied in more detail.

## Supplementary Information

**Additional file 1.** Searchteims and strategies.

**Additional file 2.** Listof excluded studies with reasons for exclusion.

## Data Availability

All data analyzed during this study are included in this published article.

## Abbreviations

AR: Allergic rhinitis
CNKI: China National Knowledge Infrastructure
VIP: China Science and Technology Journal Database
OR: Odds ratio
*95**% CI*: 95% Confidence interval
DOHaD: Developmental Origins of Health and Diseases
PICO: Patient, Indicator, Control group and outcome
SPT: Skin Prick Test
ISAAC: International study of asthma and allergies in childhood
NOS: The New Castle-Ottawa Scale
AHRQ: Agency for Healthcare Research and Quality
