# Supplementary material for: Early exposure to infections increases the risk of allergic rhinitis—a systematic review and meta-analysis
Source: BMC Pediatr. 2023 Mar 1;23:96. doi: 10.1186/s12887-023-03870-0 (PMC9976500; doi:10.1186/s12887-023-03870-0)
Supplement: Supplementary file 1 — Additional file 1. Searchteims and strategies. [file 12887_2023_3870_MOESM1_ESM.docx]

**Search teims and strategies**

**①Pubmed：**

#1 "Infections"[Mesh]

#2 ("Infection"[Title/Abstract] AND "Infestation"[Title/Abstract]) OR ("Infestation"[Title/Abstract] AND "Infection"[Title/Abstract]) OR ("Infections"[Title/Abstract] AND "Infestations"[Title/Abstract]) OR ("Infestations"[Title/Abstract] AND "Infections"[Title/Abstract]) OR "Infection"[Title/Abstract]

#3 #1 OR #2

#4 "rhinitis, allergic"[MeSH Terms] OR "rhinitis, allergic, seasonal"[MeSH Terms]

#5 Allergic Rhinitides[Title/Abstract]) OR (Rhinitides, Allergic[Title/Abstract])) OR (Allergic Rhinitis[Title/Abstract])) OR (Seasonal Allergic Rhinitis[Title/Abstract])) OR (Allergic Rhinitides, Seasonal[Title/Abstract])) OR (Allergic Rhinitis, Seasonal[Title/Abstract])) OR (Rhinitides, Seasonal Allergic[Title/Abstract])) OR (Rhinitis, Seasonal Allergic[Title/Abstract])) OR (Seasonal Allergic Rhinitides[Title/Abstract])) OR (Pollen Allergy[Title/Abstract])) OR (Allergies, Pollen[Title/Abstract])) OR (Allergy, Pollen[Title/Abstract])) OR (Pollen Allergies[Title/Abstract])) OR (Pollinosis[Title/Abstract])) OR (Pollinoses[Title/Abstract])) OR (Hay Fever[Title/Abstract])) OR (Fever, Hay[Title/Abstract])) OR (Hayfever[Title/Abstract])

#6 #4 OR #5

#7 "antenatal"[Title/Abstract] OR "Prenatal"[Title/Abstract] OR "Pregnancy"[Title/Abstract] OR "Pregnant"[Title/Abstract] OR "Perinatal"[Title/Abstract] OR "Gestational"[Title/Abstract] OR "Maternal"[Title/Abstract] OR "Mother"[Title/Abstract] OR "Newborn"[Title/Abstract] OR "Infant"[Title/Abstract] OR "early life"[Title/Abstract] OR "toddler"[Title/Abstract]

#8 #3 AND #6 AND #7

②**Web of science:**

#1 TS=(Rhinitis, Allergic OR Rhinitis, Allergic, Seasonal OR Allergic Rhinitides OR Rhinitides, Allergic OR Allergic Rhinitis OR Seasonal Allergic Rhinitis OR Allergic Rhinitides, Seasonal OR Allergic Rhinitis, Seasonal OR Rhinitides, Seasonal Allergic OR rhinoconjunctivitis OR Rhinitis, Seasonal Allergic OR Seasonal Allergic Rhinitides OR Pollen Allergy OR Allergies, Pollen OR Allergy, Pollen OR Pollinosis OR Pollinoses OR Hay Fever OR Fever, Hay OR Hayfever)

#2 TS=(Infections OR Infection and Infestation OR Infestation and Infection OR Infections and Infestations OR Infestations and Infections OR Infection)

#3 TS=(antenatal OR Prenatal OR Pregnancy OR Pregnant OR Perinatal OR Gestational OR Maternal OR Mother OR Newborn OR Infant OR Early life OR toddler OR Newborn OR Infant OR Early life OR toddler)

#4 #1 AND #2 AND #3

**③Cochrane:**

#1 MeSH descriptor: [Infections] explode all trees

#2 (Infection and Infestation):ab,ti,kw OR (Infestation and Infection):ab,ti,kw OR (Infections and Infestations):ab,ti,kw OR (Infestations and Infections):ab,ti,kw OR (Infection):ab,ti,kw

#3 #1 or #2

#4 MeSH descriptor: [Rhinitis, Allergic] explode all trees

#5 (Rhinitis, Allergic, Seasonal):ab,ti,kw OR (Allergic Rhinitides):ab,ti,kw OR (Rhinitides, Allergic):ab,ti,kw OR (Allergic Rhinitis):ab,ti,kw OR (Seasonal Allergic Rhinitis):ab,ti,kw OR (Allergic Rhinitides, Seasonal):ab,ti,kw OR (Allergic Rhinitis, Seasonal):ab,ti,kw OR (Rhinitides, Seasonal Allergic):ab,ti,kw OR (Rhinitis, Seasonal Allergic):ab,ti,kw OR (Seasonal Allergic Rhinitides):ab,ti,kw OR (rhinoconjunctivitis):ab,ti,kw OR (Pollen Allergy):ab,ti,kw OR (Allergies, Pollen):ab,ti,kw OR (Allergy, Pollen):ab,ti,kw OR (Pollinosis):ab,ti,kw OR (Pollinoses):ab,ti,kw OR (Hay Fever):ab,ti,kw OR (Fever, Hay):ab,ti,kw OR (Hayfever):ab,ti,kw

#6 #4 or #5

#7 (antenatal):ab,ti,kw OR (Prenatal):ab,ti,kw OR (Pregnancy):ab,ti,kw OR (Pregnant):ab,ti,kw OR (Perinatal):ab,ti,kw OR (Gestational):ab,ti,kw OR (Maternal):ab,ti,kw OR (Mother):ab,ti,kw OR (Newborn):ab,ti,kw OR (Infant):ab,ti,kw OR (Early life):ab,ti,kw OR (toddler):ab,ti,kw

#8 #3 and #6 and #7

**④Embase:**

#1 'rhinitis, allergic':ab,ti OR 'rhinitis, allergic, seasonal':ab,ti OR 'allergic rhinitides':ab,ti OR 'rhinitides, allergic':ab,ti OR 'allergic rhinitis':ab,ti OR 'seasonal allergic rhinitis':ab,ti OR 'allergic rhinitides, seasonal':ab,ti OR 'allergic rhinitis, seasonal':ab,ti OR 'rhinitides, seasonal allergic':ab,ti OR 'rhinitis, seasonal allergic':ab,ti OR 'seasonal allergic rhinitides':ab,ti OR 'pollen allergy':ab,ti OR 'allergies, pollen':ab,ti OR 'allergy, pollen':ab,ti OR 'pollinosis':ab,ti OR 'pollinoses':ab,ti OR 'hay fever':ab,ti OR 'fever, hay':ab,ti

#2 'infections':ab,ti OR 'infection and infestation':ab,ti OR 'infestation and infection':ab,ti OR 'infections and infestations':ab,ti OR 'infestations and infections':ab,ti OR 'infection':ab,ti

#3 'antenatal':ab,ti OR 'prenatal':ab,ti OR 'pregnancy':ab,ti OR 'pregnant':ab,ti OR 'perinatal':ab,ti OR 'gestational':ab,ti OR 'maternal':ab,ti OR 'mother':ab,ti OR 'newborn':ab,ti OR 'infant':ab,ti OR 'early life':ab,ti OR 'toddler':ab,ti

#4 #1 AND #2 AND #3

The following four are Chinese databases, which are actually retrieval strategies in Chinese.

⑤**Sinomed:**

#1" rhinitis, allergic "[Unweighted: Extended]

#2" rhinitis, allergic, seasonal" [Unweighted: Extended].

#3 allergic rhinitides or rhinitides, allergic or hay fever or fever, hay or seasonal allergic rhinitis

#4 #1 OR #2 OR #3

#5"infections"[ Unweighted: Extended]

#6 Infection during pregnancy or prenatal infection or perinatal infection or infection in pregnant woman or infection in mother during pregnancy or perinatal infection or maternal infection or neonatal infection or infancy infection or infant infection or early life infection.

#7 #5 OR #6

#8 #4 AND #7

**⑥CNKI:**

#1 Topics:rhinitis, allergic, seasonal or rhinitis, allergic, seasonal or allergic rhinitides or rhinitides, allergic or hay fever or fever, hay or seasonal allergic rhinitis or allergic disease.

#2 Title/Abstract/Keywords:Infection during pregnancy or prenatal infection or perinatal infection or infection in pregnant woman or infection in mother during pregnancy or perinatal infection or maternal infection or neonatal infection or infancy infection or infant infection or early life infection.

#3 #1 and #2

**⑦WangFang:**

Topics:( Infection during pregnancy or prenatal infection or perinatal infection or infection in pregnant woman or infection in mother during pregnancy or perinatal infection or maternal infection or neonatal infection or infancy infection or infant infection or early life infection.) and Topics:( rhinitis, allergic, seasonal or rhinitis, allergic, seasonal or allergic rhinitides or rhinitides, allergic or hay fever or fever, hay or seasonal allergic rhinitis or allergic disease)

⑧**VIP:**

Topics:( Infection during pregnancy or prenatal infection or perinatal infection or infection in pregnant woman or infection in mother during pregnancy or perinatal infection or maternal infection or neonatal infection or infancy infection or infant infection or early life infection.) and Topics:( rhinitis, allergic, seasonal or rhinitis, allergic, seasonal or allergic rhinitides or rhinitides, allergic or hay fever or fever, hay or seasonal allergic rhinitis or allergic disease)
