# Supplementary material for: Early exposure to infections increases the risk of allergic rhinitis—a systematic review and meta-analysis
Source: BMC Pediatr. 2023 Mar 1;23:96. doi: 10.1186/s12887-023-03870-0 (PMC9976500; doi:10.1186/s12887-023-03870-0)
Supplement: Supplementary file 2 — Additional file 2. Listof excluded studies with reasons for exclusion. [file 12887_2023_3870_MOESM2_ESM.docx]

List of excluded studies with reasons for exclusion

| **References** | **Reasons for exclusion** |
| --- | --- |
| [1] Amberbir A, Medhin G, Erku W, et al. Effects of helicobacter pylori and intestinalmicroflora on the risk of allergic disease and sensitization in young children[J].AmericanJournal of Respiratory and Critical Care Medicine, 2010, 181(1). | Study subjects or exposure factors or outcomes was inconsistent |
| [2] Bager P, Westergaard T, Rostgaard K, et al. Age at childhood infections and risk of atopy[J]. Thorax, 2002, 57(5): 379-82. | √ |
| [3] Cohet C, Cheng S, Macdonald C, et al. Infections, medication use, and the prevalence of symptoms of asthma, rhinitis, and eczema in childhood[J]. Journal of epidemiology and community health, 2004, 58(10): 852-7. | √ |
| [4] Hardjojo A, Goh A, Shek LPC, et al. Rhinitis in the first 18 months of life: Exploring the role of respiratory viruses[J]. Pediatric Allergy and Immunology, 2015, 26(1): 25-33. | √ |
| [5] Mehanna N, Mohamed N, Wordofa M, et al. Allergy-related disorders (ARDs) among Ethiopian primary school-aged children: Prevalence and associated risk factors[J]. PloS one, 2018, 13(9). | √ |
| [6] Mommers M, Swaen GMH, Weishoff-Houben M, et al. Childhood infections and risk of wheezing and allergic sensitisation at age 7-8 years[J]. European Journal of Epidemiology, 2004, 19(10): 945-51. | √ |
| [7] Pelosi U, Porcedda G, Tiddia F, et al. The inverse association of salmonellosis in infancy with allergic rhinoconjunctivitis and asthma at school-age: a longitudinal study[J]. Allergy, 2005, 60(5): 626-30. | √ |
| [8] Floistrup H, Swartz J, Bergstrom A, et al. Allergic disease and sensitization in Steiner school children[J]. Journal of Allergy and Clinical Immunology, 2006, 117(1): 59-66. | √ |
| [9] Sidorchuk A, Lagarde F, Pershagen G, et al. Epstein-Barr virus infection is not associated with development of allergy in children[J]. Pediatric Infectious Disease Journal, 2003, 22(7): 642-7. | √ |
| [10] Tamay Z, Akcay A, Ones U, et al. Prevalence and risk factors for allergic rhinitis in primary school children[J]. International Journal of Pediatric Otorhinolaryngology, 2007, 71(3): 463-71. | √ |
| [11] Villarreal AB, Aguirre LHS, Rojo MMT, et al. Risk factors for asthma in school children from Ciudad Juarez, Chihuahua[J]. Journal of Asthma, 2003, 40(4): 413-23. | √ |
| [12] Vörös K, Bobvos J, Varró JM, et al. Impacts of long-term ragweed pollen load and other potential risk factors on ragweed pollen allergy among schoolchildren in Hungary[J]. Annals of agricultural and environmental medicine : AAEM, 2018, 25(2): 307-13. | √ |
| [13] Williams LK, Peterson EL, Ownby DR, et al. The relationship between early fever and allergic sensitization at age 6 to 7 years[J]. Journal of Allergy and Clinical Immunology, 2004, 113(2): 291-6. | √ |

| **References** | **Reasons for exclusion** |
| --- | --- |
| [14] Kurosaka F, Terada T, Tanaka A, et al. Risk factors for wheezing, eczema and rhinoconjunctivitis in the previous 12 months among six-year-old children in Himeji City, Japan: food allergy, older siblings, day-care attendance and parental allergy history[J]. Allergology international : official journal of the Japanese Society of Allergology, 2011, 60(3): 317-30. | Study subjects or exposure factors or outcomes was inconsistent |
| [15] Loo EXL, Liew TM, Yap GC, et al. Trajectories of early-onset rhinitis in the Singapore GUSTO mother-offspring cohort[J]. Clinical and experimental allergy : journal of the British Society for Allergy and Clinical Immunology, 2021, 51(3): 419-29. | √ |
| [16] Mommers M, Thijs C, Stelma F, et al. Timing of infection and development of wheeze, eczema, and atopic sensitization during the first 2 yr of life: The KOALA Birth Cohort Study[J]. Pediatric Allergy and Immunology, 2010, 21(6): 983-9. | √ |
| [17] Sun YX, Sundell J. Early Daycare Attendance Increase the Risk for Respiratory Infections and Asthma of Children[J]. Journal of Asthma, 2011, 48(8): 790-6. | √ |

| **References** | **Reasons for exclusion** |
| --- | --- |
| [1] Arruda LK, Solé D, Baena-Cagnani CE, et al. Risk factors for asthma and atopy[J]. Current opinion in allergy and clinical immunology, 2005, 5(2): 153-9. | Systematic review or review |
| [2] Daley D. The evolution of the hygiene hypothesis: the role of early-life exposures to viruses and microbes and their relationship to asthma and allergic diseases[J]. Current opinion in allergy and clinical immunology, 2014, 14(5): 390-6. | √ |
| [3] Gangal SV, Chowgule R. Infections in early life and susceptibility to allergic diseases: relevance of hygiene hypothesis[J]. Current Science, 2009, 96(6): 784-93. | √ |
| [4] Hammersley VS, Sheikh A. Early life infections and the risk of hay fever[J]. Primary Care Respiratory Journal, 2008, 17(3): 194. | √ |
| [5] Hersoug LG. A reformulation of the hygiene hypothesis: Maternal infectious diseases confer protection against asthma in the infant[J]. Medical Hypotheses, 2006, 67(4): 717-20. | √ |
| [6] Howarth PH. Is allergy increasing? early life influences[J]. Clinical and Experimental Allergy, 1998, 28: 2-7. | √ |
| [7] Kim DS, Drake-Lee B. Infection, allergy and the hygiene hypothesis: historical perspective[J]. Journal of Laryngology and Otology, 2003, 117(12): 946-50. | √ |
| [8] Macintyre EA, Heinrich J. Otitis media in infancy and the development of asthma and atopic disease[J]. Current allergy and asthma reports, 2012, 12(6): 547-50. | √ |
| [9] Miftahussurur M, Nusi IA, Graham DY, et al. Helicobacter, Hygiene, Atopy, and Asthma[J]. Frontiers in Microbiology, 2017, 8. | √ |
| [10] Okamoto Y, Sakurai D, Horiguchi S. Allergic rhinitis in children: Environmental factors[J]. Clinical and Experimental Allergy Reviews, 2004, 4(1): 9-14. | √ |
| [11] Poddighe D. "Home environment and diseases in early life are associated with allergic rhinitis": Role of respiratory infections and passive smoke exposure in infancy[J]. International Journal of Pediatric Otorhinolaryngology, 2019, 125: 133-. | √ |

| **References** | **Reasons for exclusion** |
| --- | --- |
| [12] Quiros AB, Sanz EA. Early infections and later allergic diseases[J]. Allergologia et immunopathologia, 2009, 37(6): 279-80. | Systematic review or review |
| [13] Renz H, Herz U. The bidirectional capacity of bacterial antigens to modulate allergy and asthma[J]. European Respiratory Journal, 2002, 19(1): 158-71. | √ |
| [14] Tay CJX, Ta LH, Yeong YXO, et al. Role of Upper Respiratory Microbiota and Virome in Childhood Rhinitis and Wheeze: Collegium Internationale Allergologicum Update 2021[J]. International Archives of Allergy and Immunology, 2021, 182(4): 265-76. | √ |
| [15] Varner AE. The increase in allergic respiratory diseases - Survival of the fittest?[J]. Chest, 2002, 121(4): 1308-16. | √ |
| [16] Liu Kai, Zhang Jian. Chlamydia pneumoniae infection and allergic rhinitis. [J]. International Journal of Otolaryngology-Head and Neck Surgery, 2013, 37(2): 85-7. | √ |
| [17] He Yun, Hong Suling. Effects of early infection on TH1/TH2 immune balance in allergic rhinitis [J]. Journal of Chongqing Medical University, 2006, 31(z1): 17-20. | √ |
| [18] Fan Yongchen. Allergic rhinitis and related diseases in children [J]. Chinese Journal of Practical Pediatrics, 2002, 17(7): 435-7. | √ |

| **References** | **Reasons for exclusion** |
| --- | --- |
| [1] Dana VG, Fazlollahi MR, Abbassi JM, et al. Study of the allergic symptoms prevalence and some relevant factors among school children in tehran[J]. Journal of Allergy and Clinical Immunology, 2017, 139(2): AB26. | Unable to obtain full text or effect value |
| [2] Figueiredo EA, Bettiol H, Gutierrez M, et al. Prevalence and risk factors associated with allergic sensitisation, rhinitis and eczema among children: Comparison of two birth cohorts from different cities in Brazil[J]. Allergy: European Journal of Allergy and Clinical Immunology, 2013, 68: 53-4. | √ |
| [3] Huang YW, Tsai HJ, Tsai YT, et al. Enterovirus infection in early life and risk of developing allergic diseases in children[J]. Allergy: European Journal of Allergy and Clinical Immunology, 2016, 71: 390. | √ |
| [4] Lubyayi L, Mpairwe H, Nkurunungi G, et al. Infection-exposure in infancy is associated with reduced allergy-related disease in later childhood in a Ugandan cohort[J]. Elife, 2021, 10. | √ |
| [5] Matheson MC, Walters EH, Simpson JA, et al. Relevance of the hygiene hypothesis to early vs. late onset allergic rhinitis[J]. Clinical and Experimental Allergy, 2009, 39(3): 370-8. | √ |
| [6] Meyer KE, Arguelles LM, Kumar R, et al. Early life antibiotic use and allergic rhinitis[J]. Journal of Allergy and Clinical Immunology, 2009, 123(2): S272. | √ |
| [7] Pekkanen J, Remes S, Kajosaari M, et al. Infections in early childhood and risk of atopic disease[J]. Acta Paediatrica, 1999, 88(7): 710-4. | √ |
| [8] Zutavern A, Von Klot S, Gehring U, et al. Pre-natal and post-natal exposure to respiratory infection and atopic diseases development: a historical cohort study[J]. Respiratory Research, 2006, 7 | √ |

| **References** | **Reasons for exclusion** |
| --- | --- |
| [1] Ramírez-Del-Pozo ME, Gómez-Vera J, López-Tiro J. [Risk factors associated with the development of atopic march. Case-control study][J]. Revista alergia Mexico (Tecamachalco, Puebla, Mexico : 1993), 2012, 59(4): 199-203. | Not Chinese or English Studies |
| [2] Kurz H, Riedler J. [An increase in allergic diseases in childhood--current hypotheses and possible prevention][J]. Wiener medizinische Wochenschrift (1946), 2003, 153(3-4): 50-8. | √ |
| [3] Gniazdowska B, Jefimow A. [Epidemiologic studies on allergic diseases among rural and urban school children in Poland][J]. Polski tygodnik lekarski (Warsaw, Poland : 1960), 1990, 45(42-44): 855-60. | √ |
|  |  |
| [1] Shim JY, Kim HB, Lee SY, et al. Effects of early measles on later rhinitis and bronchial hyperresponsiveness[J]. Annals of Allergy Asthma & Immunology, 2010, 105(1): 43-9. | Conference Abstract |
| [2] Ye J, Kang X, Tian T. Disease in the first 6 months of life and environmental factors associated with allergic rhinitis[J]. Allergy: European Journal of Allergy and Clinical Immunology, 2018, 73: 568-71. | √ |
|  |  |
| [1] Ryozawa M, Matsubara T, Ichiyama T, et al. Clinical sepsis in neonates is responsible for the lower prevalence of developing allergy[J]. Pediatrics international : official journal of the Japan Pediatric Society, 2007, 49(1): 15-8. | Calculations were required to obtain crude OR values, and the number of study participants was very small. |
| [2] Yildizdas HY, Ozcan A, Sertdemir Y, et al. Effect of healthcare associated infections and broad spectrum antibiotic use in newborn period on development of asthma, allergic rhinitis and atopic dermatitis in early childhood[J]. Cukurova Medical Journal, 2017, 42(1): 132-9. | √ |
|  |  |
| [3] Shim JY, Kim HB, Lee SY, et al. Effects of early measles on later rhinitis and bronchial hyperresponsiveness[J]. Annals of Allergy Asthma & Immunology, 2010, 105(1): 43-9. | Measles infection(only one) |
